# Prevalence of Visual Impairment in School Children

**DOI:** 10.18502/jovr.v15i1.5968

**Published:** 2020-02-02

**Authors:** Reza Gharebaghi, Fatemeh Heidary

**Affiliations:** ^1^International Virtual Ophthalmic Research Center (IVORC), Tehran, Iran; ^2^Department of Ophthalmology, Faculty of Medicine, Infectious Ophthalmic Research Center, Ahvaz Jundishapur University of Medical Sciences, Ahvaz, Iran

Dear Editor,

The recent published article by Talebnejad et al sheds light on the prevalence of visual impairments and their associated determinants in school children in Shiraz, south of Iran.^[1]^ We hope this imperative report spurs community action regarding the importance of population-based studies and provides basic information about the targeted prevention programs for children. Of course, the results should be compared with the outcomes of the Iranian State Welfare Organization (ISWO) visual screening programs for children.

We previously published two major studies in this field that derived from the same sample size of the current study but unfortunately were not cited in the recent publication. Firstly, we included 262 eyes of Iranian primary school children in Shiraz between 6 and 13 years of age and found that intraocular pressure (IOP) and central corneal thickness (CCT) in healthy school children were positively correlated.^[2]^ Secondly, in another published study, we analyzed the relationship between different parameters of Ocular Response Analyzer (ORA) and Corvis ST (CST) in school-aged children in Shiraz, and the relationship between parameters of these two instruments versus the IOP measured by Goldmann applanation tonometer (GAT) was evaluated. We found the highest IOP overestimation by CST and the lowest by corneal-compensated IOP (IOP-CC) compared with GAT. Overall, either low positive correlation or negligible correlation was found among the IOP measurements by three instruments.^[3]^ Furthermore, there are several studies discussing the effect of race on the biometric data of ocular structures. This is an essential issue since recently in a meta-analysis, the authors showed that corneal thickness is thinner in children originating from mixed Malay-Indian race than in most other locations.^[4]^ Lastly, a few publications have revealed that age may affect the ocular biometrics.^[5]^ Therefore, we would

suggest that the authors reiterate all measurements as a cohort analysis since almost five years have passed since the initiation of the study in 2015.

## References

[1] Talebnejad M., Nowroozzadeh M., Mahdaviazad H., Khalili M., Masoumpour M., Keshtkar M., Mohammadi E., Tajbakhsh Z. (2018). The Shiraz pediatric eye study; a population based survey of school age children: Rationale, design and baseline characteristics. *Journal of Ophthalmic & Vision Research*.

[2] Nejabat M., Heidary F., Talebnejad M. R., Salouti R., Nowroozzadeh M. H., Masoumpour M., Mahdaviazad H., Tajbakhsh Z., Keshtkar M., Jamali H., Khalili M. R., Movahedan H., Roustaei N., Gharebaghi R. (2016). Correlation Between Intraocular Pressure and Central Corneal Thickness in Persian Children. *Ophthalmology and Therapy*.

[3] (2018). Comparison among Ocular Response Analyzer, Corvis ST and Goldmann applanation tonometry in healthy children. *International Journal of Ophthalmology*.

[4] Farvardin M., Heidary F., Sayehmiri K., Gha-Rebaghi R., Jabbarvand Behrooz M. (2017). A comprehensive meta-analysis on intra ocular pressure and central corneal thickness in healthy children. *Iranian Journal of Public Health*.

[5] Doughty M. J., Zaman M. L. (2000). Human corneal thickness and its impact on intraocular pressure measures: a review and meta-analysis approach. *Survey of Ophthalmology*.

